# MRI-Based Demonstration of the Normal Glymphatic System in a Human Population: A Systematic Review

**DOI:** 10.3389/fneur.2022.827398

**Published:** 2022-05-25

**Authors:** Min Kyoung Lee, Se Jin Cho, Yun Jung Bae, Jong-Min Kim

**Affiliations:** ^1^Department of Radiology, College of Medicine, Yeouido St. Mary's Hospital, The Catholic University of Korea, Soeul, South Korea; ^2^Department of Radiology, Seoul National University Bundang Hospital, Seoul National University College of Medicine, Seongnam, South Korea; ^3^Department of Neurology, Seoul National University Bundang Hospital, Seoul National University College of Medicine, Seongnam, South Korea

**Keywords:** glymphatic system, MRI, CSF, systematic review, human population

## Abstract

**Background:**

The glymphatic system has been described as one that facilitates the exchange between the cerebrospinal fluid (CSF) and interstitial fluid, and many recent studies have demonstrated glymphatic flow based on magnetic resonance imaging (MRI). We aim to systematically review the studies demonstrating a normal glymphatic flow in a human population using MRI and to propose a detailed glymphatic imaging protocol.

**Methods:**

We searched the MEDLINE and EMBASE databases to identify studies with human participants involving MRI-based demonstrations of the normal glymphatic flow. We extracted data on the imaging sequence, imaging protocol, and the targeted anatomical structures on each study.

**Results:**

According to contrast-enhanced MRI studies, peak enhancement was sequentially detected first in the CSF space, followed by the brain parenchyma, the meningeal lymphatic vessel (MLV), and, finally, the cervical lymph nodes, corresponding with glymphatic flow and explaining the drainage into the MLV. Non-contrast flow-sensitive MRI studies revealed similar glymphatic inflow from the CSF space to the brain parenchyma and efflux of exchanged fluid from the brain parenchyma to the MLV.

**Conclusion:**

We may recommend T1-weighted contrast-enhanced MRI for visualizing glymphatic flow. Our result can increase understanding of the glymphatic system and may lay the groundwork for establishing central nervous system fluid dynamic theories and developing standardized imaging protocols.

## Introduction

The lymphatic system plays a role in tissue homeostasis, interstitial fluid (ISF) clearance, and immune control (1). Historically, the central nervous system (CNS) was thought to lack a lymphatic system (1). However, this assumption has been doubted due to the discovery of interstitial solute clearance within the CNS and the immune cells which should be circulated from the lymphatic system; such contradictions have led to many studies investigating the possibility of a lymphatic-like system in the CNS (1). As a result, Illiff et al. identified a term of a lymphatic-like system in the CNS responsible for facilitating exchange between the cerebrospinal fluid (CSF) and ISF *via* perivascular water flux, which is now called “glymphatic system” (2–4). Many following studies have evaluated glymphatic system according to their hypothesis regarding perivascular water influx and have suggested that this system could not only clear interstitial solute from CNS, but may also maintain extracellular fluid homeostasis and support the immune system within the CNS (1, 5, 6).

Most existing studies of the glymphatic system have been performed using fluorescent tracers (3, 4, 7). The animal-based studies (3, 4, 6, 7) have demonstrated that the glymphatic clearance flow starts within the CSF space, followed by transport into the brain parenchyma, where fluid exchange between subarachnoid CSF and perivascular ISF occurs. The exchanged fluid flows to the meningeal lymphatic vessel (MLV) and is ultimately drained to the cervical lymph nodes (LNs; Supplementary Figure 1). Subsequently, since radiotracer studies are limited in the human population, many recent studies have endeavored to reveal the presence of the glymphatic system in humans using diverse magnetic resonance image (MRI)-based techniques. As a result, they have succeeded in demonstrating the glymphatic flow in the human population (8–17), and some even have found that dysfunction of the glymphatic system can be an underlying pathophysiologic mechanism for natural brain aging and various CNS diseases (2, 18–20). However, there is still a lack of systematic review of these glymphatic studies, particularly focusing on the MRI-based publications demonstrating the normal glymphatic system in human participants. Even though the glymphatic hypothesis is still under discussion and some recent studies have argued that not only perivascular convective flow but also solute transfer within the extracellular space play an important role in the CNS fluid movement (21, 22), the review of the glymphatic imaging studies dealing with the glymphatic hypothesis could help to establish the concept of fluid movement dynamics in the CNS. In this study, we aimed to provide a review of the studies that used MRI techniques to measure and visualize the CNS fluid flow compatible with glymphatic hypothesis. We reviewed the imaging sequence, the imaging protocol, including the contrast agent injection protocol and acquisition time points on dynamic contrast-enhanced scanning, and the targeted anatomical structures adopted in each study.

## Materials and Methods

### Literature Search

Our search included studies that investigated the presence of the glymphatic flow in a normal population. A systematic literature search was performed using the international databases of MEDLINE, EMBASE, and Cochrane Library. The search terms were ([glymphatic] OR [meningeal lymphatic] OR [CNS lymphatics] OR [lymphatics of CNS] OR [cranial lymphatic]) AND ([“magnetic resonance imaging”] OR [“MR imaging”] OR [MRI]). We searched for original studies published in full up to December 23, 2021. To expand the search, the bibliographies of relevant articles were screened to identify other appropriate articles.

### Inclusion Criteria

Studies were included if they met the following criteria: (1) they involved study participants who were healthy human volunteers who agreed to submit to brain image or those who had no CNS disease other than suspicious CSF leakage or endolymphatic hydrops; (2) studies which employed MRI; and (3) their results demonstrated the presence of the glymphatic system.

### Exclusion Criteria

Studies or subsets of studies were eliminated if they met the following conditions: (1) they were case reports with a sample size of 10 or fewer patients; (2) they were editorials, letters, abstracts, systematic reviews/meta-analyses, consensus statements, guidelines, or review articles; (3) they included animal studies of the glymphatic system; (4) they did not focus on the visualization/demonstration of the glymphatic system; (5) they were articles that had(or were suspected of having) overlapping populations; or (6) they involved study participants with neurodegenerative diseases, hydrocephalus, traumatic brain injury, cerebrovascular disease, cerebral demyelinating disease, cerebral metabolic disease, epilepsy, and/or solid brain tumor.

### Data Extraction

We extracted the following data: (1) the study characteristics including the author, year of publication, institution, country of origin, enrollment period, study design (prospective vs. retrospective), number of participants, mean participant age, male-to-female ratio, inclusion and exclusion criteria, and rationale of inclusion for each study; (2) the MRI sequence used for the glymphatic assessment: contrast-enhanced MRI using contrast agent injection vs. non-contrast imaging (flow-sensitive MRI), type of contrast material and concentration, and the method of contrast injection in the case of contrast-enhanced MRI (intrathecal vs. intravenous), acquisition time-points for dynamic contrast-enhanced MRI, and MRI scanning parameters (MR machine vendor, magnetic field strength [Tesla (T)], pulse sequences, repetition time, echo time, matrix size, field-of-view, slice thickness, and scan time); (3) the analytical method used in the glymphatic assessment: method of assessment (subjective visual assessment vs. objective signal quantification), targeted anatomical structures for the measurement, and glymphatic measurement targets (time-point dynamic enhancement curve and peak enhancement time in the contrast-enhanced MRI vs. flow change and direction in the flow-sensitive MRI). With regard to the targeted anatomical structures for the glymphatic system and flow, we considered four structures in particular, including the CSF space, brain parenchyma, MLV, and cervical LN. We defined the CSF space as the subarachnoid space near the brain parenchyma. The ISF within the brain parenchyma was defined as the brain parenchyma. The perivenous efflux drained to the MLV, and the fluid finally flowed to the cervical LNs (Supplementary Figure 1). Therefore, we included MLV and cervical LNs within the targeted anatomical structures. Two radiologists (MKL and SJC) with seven years of experience in brain imaging independently searched the literature and selected appropriate studies. Any discrepancy between the two readers was solved by consensus.

### Quality Assessment

Two reviewers independently extracted the data and performed a quality assessment using the RoBANS tool for nonrandomized controlled trials (23).

## Results

### Literature Search

**Figure 1:**
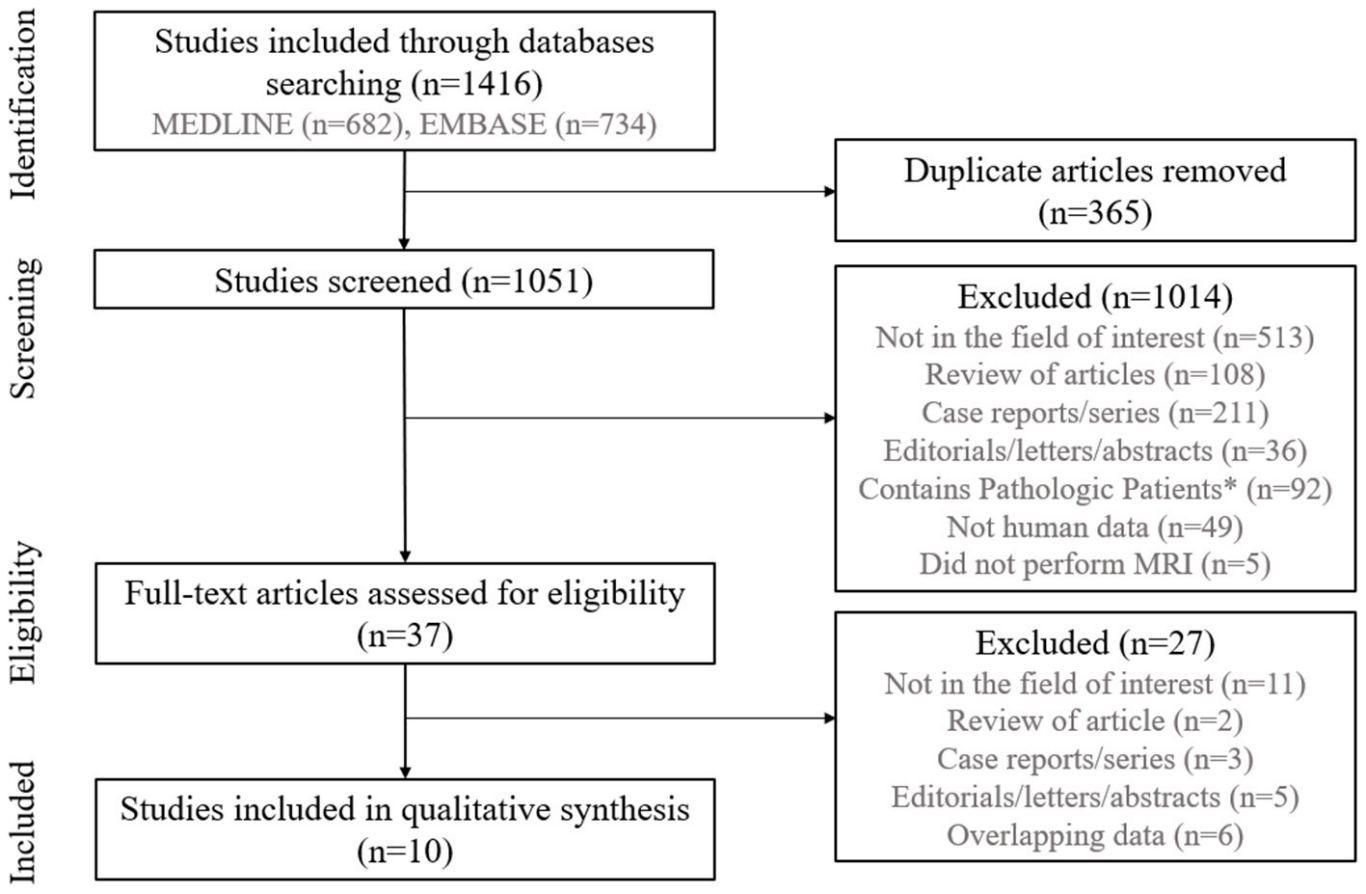
Flow diagram of the study selection process. *Patients with neurodegenerative diseases, hydrocephalus, traumatic brain injury, cerebrovascular disease, cerebral demyelinating disease, cerebral metabolic disease, epilepsy, and/or solid brain tumors were excluded.

The study selection process is illustrated in Figure 1. We identified 1,416 studies in our initial systematic search of the MEDLINE and EMBASE databases. No relevant trial was identified in the Cochrane Library. After removing 365 duplicates, screening the remaining 1,051 titles and abstracts yielded 37 potentially eligible articles. No additional articles were identified from the bibliographies of those articles. After full-text reviews of the 37 provisionally eligible articles, 27 were excluded for the following reasons: 11 did not focus on demonstration of the glymphatic system (24–34), two were reviews of articles (35, 36), three were case reports or series (37–39), five were abstracts (40–44), and six included or were suspected of including overlapping populations (45–50). Finally, our study included 10 studies were included in our qualitative systematic review (8–17).

### Characteristics of the Included Studies

Table 1 shows the clinical characteristics of the 10 included studies. The total number of participants in these studies was 274, with the sample sizes of individual studies ranging from 5 to 190 participants. The age of each study ranged from 14 to 81 years. Seven studies included more women than men (8–11, 13, 16, 17), whereas two studies had more men than women (12, 14); the remaining one study did not report sex distribution (15). Seven studies employed a prospective design (8–12, 16, 17), whereas the other three studies were retrospective in nature (13–15). Among the selected studies, four contained healthy participants; therefore, we included all of the participants from those studies (8, 11, 12, 15). Six studies investigated both healthy participants and patients with neurological disorders; among patients with neurologic disorders, we only included those who underwent intrathecal contrast-enhanced MRI for clinically suspicious spontaneous CSF leakage and those who underwent a 4-h-delayed intravenous contrast-enhanced MRI for the evaluation of endolymphatic hydrops in the absence of other CNS pathology (9, 10, 13, 14, 16, 17). The detailed exclusion criteria for each of the studies are described in the footnote of Table 1.

**Table 1:** The clinical characteristics of the included studies.

**Group**	**Source**	**Affiliation**	**Enrollment period**	**Study Design**	**Participant (n)**	**Mean agein years ±SD (range)**	**Male: Female**	**Inclusion/ Exclusion criteria**	**Rationale of inclusion**
Contrast-enhanced MRI group	Absinta M et al. (8)	National Institutes of Health, USA	NA	Pros.	5	NA (28-53)	2:3	In). Healthy participants/ Ex). NA	Healthy participants
	Eide PK et al. (9)	Oslo University Hospital-Rikshospitalet, Norway	NA	Pros.	16	36.1 ± 11.7 (19-54)	2:14	In). CSF disorder (various) with a deep cervical lymph node with size > 1.5 cm^†^/Ex). H/O hypersensitive reactions to contrast media, H/O severe allergic reactions, renal dysfunction, pregnant or breastfeeding women, and age <18 YO or >80 YO	To enroll participants undergoing MRI after i.t contrast injection, to measure the signal change in the cervical LN
	Jacobsen HH et al. (10)	Oslo University Hospital, Norway	February 2016-August 2018	Pros.	10	36.9 ± 6.95 (NA)	2:8	In). CSF disorder (SIH or pineal cyst)/ Ex). H/O hypersensitivity reactions to contrast media, H/O severe allergy reactions, renal dysfunction, pregnant or breastfeeding women, and age <18 YO or >80 YO	To enroll participants with MRI after i.t contrast injection, to measure the signal change along the visual tract
	Naganawa S et al. (13)	Nagoya University Graduate School of Medicine, Japan	NA	Retro.	190	NA (14-81)	91:99	In). Endolymphatic hydrops, with 4 hours delayed MRI after an i.v. injection of gadolinium/ Ex). Brain tumor, cerebral infarctions, H/O CNS infection, and H/O recent systematic use of steroid	To enroll participants with 4 hours delay MRI after i.v. contrast injection covering the brain and neck
	Oner et al. (14)	Gazi University School of Medicine, Turkey	1998-2014	Retro.	6	39.2 ± 20.8 (15-74)	6:0	In). CSF disorder (CSF rhinorrhea or arachnoid cyst) from cohorts of intrathecal CE-MRC/ Ex). renal dysfunction, transplantation, diabetes, and malignancies	To enroll participants with MRI after i.t contrast injection
	Ringstad G et al. (16)	Oslo University Hospital-Rikshospitalet, Norway	October 2015-May 2016	Pros.	8	41.1 ± 13.0 (NA)	2:6	In). CSF disorder (CSF leakage syndrome or intracranial cyst)^†^. H/O hypersensitive reactions to contrast media, H/O severe allergy reactions, renal dysfunction, pregnant or breastfeeding women, and age <18 YO or >80 YO	To enroll participants with MRI after i.t contrast injection
	Zhou et al. (17)	Second Affiliated Hospital of Zhejiang University, China	April 2018-November 2019	Pros.	14	55.9 ± 12.7 (20-71)	6:8	In). CSF disorder (leakage) or peripheral neuropathy from cohorts of lumbar puncture and voluntary participation^†^/ Ex). H/O hypersensitivity reactions to contrast media, H/O severe allergy reactions, renal dysfunction, and pregnant or breastfeeding women	To enroll participants with MRI after i.t contrast injection
Non-contrast MRI group	Kiviniemi et al. (11)	Oulu University Hospital, Finland	NA	Pros.	9	25.67 ± 4.52 (NA)	4:5	In). Healthy participants/ Ex). NA	Healthy participants
	Kuo et al. (12)	University of Arizona, USA	NA	Pros.	6	NA (30–56)	4:2	In). Healthy participants/ Ex). NA	Healthy participants
	Rajna Z et al. (15)	Oulu University Hospital, Finland	NA	Retro.	10	58.3 ± 9.9 (NA)	NA	In). Healthy participants^†^/Ex). NA	Healthy participants

*CE-MRC, contrast-enhanced magnetic resonance cisternography; CNS, central nervous system; CSF, cerebrospinal fluid; Ex)., exclusion; H/O, history of; In)., inclusion; i.t, intrathecal; i.v.; intravenous; LN, lymph node; MRI, magnetic resonance imaging; NA, not applicable; Pros., Prospective; Retro., retrospective; SD, standard deviation; SIH, spontaneous intracranial hypotension; YO, years old*.

† *We excluded the following number of participants from these studies: Eide et al., three patients with hydrocephalus; Ringstad et al., 15 patients with hydrocephalus; Zhou et al., 23 patients with brain diseases, including cerebrovascular disease, neurodegenerative disease, hydrocephalus, encephalitis, and metabolic disease; Rajna et al., 10 patients with Alzheimer's disease*.

### *MRI* Sequence for Glymphatic Assessment

Table 2 shows the detailed MRI sequence and protocol for the glymphatic assessment used in each study. The studies were categorized into two groups, including a contrast-enhanced MRI group and a flow-sensitive MRI group, according to whether they involved contrast injection. The contrast-enhanced MRI group comprised studies whose participants underwent post-contrast MRI acquisition after intrathecal or intravenous contrast agent injection. The non-contrast MRI group comprised studies whose participants who did not undergo contrast agent injection by any route, but flow-sensitive MRI was performed, including time-of-flight MR angiograph (TOF-MRA) and phase-contrast MRI. Accordingly, the contrast-enhanced MRI group comprised seven studies that assessed the presence of the glymphatic system by detecting contrast enhancement within that system (8–10, 13, 14, 16, 17), whereas the non-contrast MRI group comprised three studies that evaluated the glymphatic system by delineating the CSF flow through that system (11, 12, 15). Among the seven studies in the contrast-enhanced MRI group, five employed intrathecal contrast injection (9, 10, 14, 16, 17), whereas two administered contrast agent *via* intravenous injection (8, 13). Most contrast agents were gadolinium-based extracellular fluid agents, including gadobutrol (8–10, 13, 16), gadopentetate dimeglumine (14), and gadodiamide (17). Four of the seven studies in the contrast-enhanced MRI group performed time-point dynamic observations within the targeted structures in the glymphatic system (9, 10, 16, 17), whereas the other three studies in the contrast-enhanced group (8, 13, 14) and all of the studies in the non-contrast MRI group (11, 12, 15) did not perform time-point dynamic observations. The details regarding the time schedules of the time-point dynamic observations are listed in Table 2.

**Table 2:** MRI sequence used for glymphatic assessment.

**Group**	**Source**	**Contrast material (concentrate)**	**Route of contrast Injection**	**Dynamic observation**	**Time schedule in dynamic observation**	**Detailed MR protocol**								
						**Vendor, machine**	**Magnetic field strength (T)**	**MR sequence**	**TR (ms)/TE (ms)**	**Matrix size**	**FOV, (mm^**2**^)**	**Slice thickness (mm)**	**Pixel/voxel size (mm)**	**Scan time, (min)**
Contrast-enhancement MRI group	Absinta et al. (8)	Gadobultrol (0.1 mmol/ml)	i.v	No	NA	Skyra, Siemens	3	T1WI (black blood,SPACE); FLAIR (SPACE)	938/22; 4,800/354	512 × 512, 320 × 320	174 × 174; 235 × 235	0.5; 1	0.3; 0.7/NA	7.8; 14
	Eide et al. (9)	Gadobultrol (1.0 mmol/ml)	i.t	Yes	Pre., 2–4, 4–6, 6–9, 24, and 48 h	Ingenia, Philips	3	T1WI	5.1/2.3	256 × 256	512 × 512	1	2/NA	6.5
	Jacobsen et al. (10)	Gadobutrol (1.0 mmol/ml)	i.t	Yes	0–20, 20–40, and 40–60 min, 1–2, 2–4, 4–6, 6–9, 24, and 48 h	Ingenia, Philips	3	T1WI	5.1/2.3	256 × 256	256 × 256	1	1/NA	6.5
	Naganawa et al. (13)	Gadobutrol (0.1 mmol/ml)	i.v	No	4 h	Skyra, Siemens	3	FLAIR (SPACE)	15,130/549	324 × 384	165 × 196	1	0.50 × 0.5/NA	10
	Oner et al. (14)	Gadopentetate dimeglumine (NA)	i.t	No	NA	Excite, GE; Verio, Siemens	1.5, 3	T1WI	550/12	288 × 160	20	5	0.070 × 0.125 /NA	NA
	Ringstad et al. (16)	Gadobutrol (1.0 mmol/ml)	i.t	Yes	Pre, 0–20, 20–40, and 40–60 min, 1–2, 2–4, 4–6, 6–9, and 24 h	Ingenia, Philips	3	T1WI	5.1/2.3	256 × 256	256 × 256	1	1/NA	6.5
	Zhou et al. (17)	Gadodiamide (0.5 mmol/ml)	i.t	Yes	4.5, 15, and 39 h	GE 750, GE	3	FLAIR (2D and 3D CUBE)	8,400/152; 5,000/131	320 × 320; 256 × 256	18 × 18; 23.5 × 23.5	3; NA	0.56/NA; 0.9/NA	NA; NA
Non-contrast MRI group	Kiviniemi et al. (11)	No	No	No	NA	Skyra, Siemens	3	MREG	100/36	64 × 64 × 64	NA	NA	0.3/0.310 × 0.311 × 0.5	10
	Kuo et al. (12)	No	No	No	NA	Skyra, Siemens	3	TOF MRA	30/4.49	160 × 160	50	1.5	NA	NA
	Rajna et al. (15)	No	No	No	NA	Skyra, Siemens	3	MREG	100/1.4	NA	NA	NA		NA

*2D, two-dimension; 3D, three-dimension; CE-MRC, contrast-enhanced MR cisternography; CE-T1WI, contrast-enhanced T1-weighted image; CUBE, a kind of high resolution 3D turbo spin echo acquisition GE; DWI, diffusion weighted image; FLAIR, fluid attenuated inversion recovery; FOV, field-of-view; i.t, intrathecal; i.v., intravenous; MREG, MR encephalography(ultra-fast 3D k-space under-sampling technique); MPRAGE, magnetization prepared rapid gradient echo as a high resolution whole brain T1-weighted imaging; NA, not applicable; ROI, region of interest; SPACE, sampling perfection with application-optimized contrasts by using different flip angle evaluation as a high resolution 3D turbo spin echo acquisition in Siemens; SSS, superior sagittal sinus; T, tesla; T1WI, T1-weighed image; T2WI, T2-weighted image; TE, echo time; TOF MRA, Time-of-Flight MR angiography; TR, repetition time*.

Of the studies in the contrast-enhanced MRI group, six performed MRI examinations using 3.0-T scanners (8–10, 13, 16, 17), whereas one study conducted MRI examinations using either 1.5- or 3.0-T scanner (14). All studies in the non-contrast MRI group performed MRI examinations using 3.0-T scanners (11, 12, 15). All studies in the contrast-enhanced MRI group evaluated contrast enhancement on conventional images, including T1-weighted or T2-weighted fluid attenuated inversion recovery (FLAIR) images (8–10, 13, 14, 16, 17). However, the imaging parameters for each sequence differed between studies. The detailed MRI parameters are described in Table 2. Regarding the three studies in the non-contrast MRI group, two utilized phase-contrast MR encephalography (MRE) for assessing CSF flow (11, 12), whereas the third performed TOF-MRA for CSF flow (Table 2).

### Analytical Method for Glymphatic Assessment

Table 3 shows the analytic method used for glymphatic assessment in each study. Six of the included studies used a method allowing for objective signal quantification by measuring the signal ratio change or flow change (9–11, 15–17), whereas three of the included studies employed a subjective visual assessment of contrast enhancement or flow change (8, 12, 13); the final study utilized both subjective and objective analytical methods (14). The targeted anatomic structures for the glymphatic measurement are described in Table 3. Among the studies in the contrast-enhanced MRI group, four studies assessed contrast signal changes in multiple locations (9, 10, 16, 17); one of the study evaluated changes at all four locations (17), one study at three locations (CSF space, brain parenchyma, and cervical LNs) (9), and two studies at two locations (CSF space and brain parenchyma) (10, 16). The two studies in the non-contrast MRI group assessed the flow-signal change at multiple locations, including the CSF space, brain parenchyma, and MLV (11, 15). The other four studies (comprising three contrast-enhanced MRI studies and one non-contrast MRI study) examined the flow-signal change at only one location; the CSF space in one study (13), the brain parenchyma in one study (14), and the MLV in two studies (8, 12). The most common locations for signal measurements were the CSF space (9–11, 13, 15–17) and the brain parenchyma (9–11, 14–17) (in seven studies each). The MLV was evaluated in five studies (8, 11, 12, 15, 17), and cervical LNs were assessed in two studies (9, 17). Time-point dynamic observations were performed in the contrast-enhanced MRI group, but not in the non-contrast MRI group. All four studies that carried out time-point dynamic observations in the contrast-enhanced MRI group assessed the change in signal ratio and detected the peak enhancement time at multiple anatomic locations (9, 10, 16, 17). As a result, the peak enhancement times following contrast injection according to the targeted structure were 4–6 h within the CSF space (range: 3–9 h), 24–48 h within the brain parenchyma (range: 15–48 h), 15 h within the MLV (range: 15–39 h), and 24–48 h within the cervical LNs (range: 24–48 h).

**Table 3:** Analytic method used in the glymphatic assessment of each sequence.

**Group**	**Source**	**Subj. vs. Obj**.	**Targeted anatomical structures**				**Time-point dynamic enhancement and peak enhancement time**	**Flow change and direction**
			**CSF**	**Parenchyma**	**MLV**	**Cervical LNs**		
Contrast-enhancement MRI group	Absinta et al. (8)	Subj.	No	No	Yes (5 of 5)	No	NA	NA
	Eide et al. (9)	Obj.	Yes (near IFG after 4–9 h in 13 of 16 individuals)	Yes (IFG (in 14 of 15)^*^, PHG, thalamus, and pons after 24–48 h)	No	Yes (LN after 24–48 h in 9 of 15^*^ individuals)	Peak glymphatic enhancement occurred within the CSF space (near IFG) and brain parenchyma (IFG) on T1WI after 4–6 h and 24–48 h	NA
	Jacobsen et al. (10)	Obj.	Yes (prechiasmatic cistern after 4–6 h)	Yes (optic nerve, optic chiasm, optic tract, and primary visual cortex after 24 h, except optic chiasm (6–9 h))	No	No	Peak glymphatic enhancement occurred within the CSF (prechiasmatic cistern) and brain parenchyma of visual pathway (optic nerve, optic tract, and primary visual cortex) on T1WI after 4–6 h and 24 h	NA
	Naganawa et al. (13)	Subj	Yes (around the cortical veins after 4 h in 155 of 190 individuals)	No	No	No	Glymphatic enhancement occurred within the CSF space around the cortical vein on FLAIR after 4 h	NA
	Oner et al. (14)	Subj. & Obj.	No	Yes (globus pallidus and dentate nucleus in 5 of 6 individuals/ Increment of signal intensity ratio within brain parenchyma on T1WI (in 6 of 6))^†^	No	No	Glymphatic enhancement occurred within the brain parenchyma (dentate nucleus and globus pallidus) on T1WI	NA
	Ringstad et al. (16)	Obj.	Yes (foramen magnum (1–2 h), pontine cistern (1–2 h), Sylvian fissure (4–6 h), 3^rd^ and 4^th^ ventricles (4–6 h), central sulcus (4–6 h), and lateral ventricle (6–9 h), and after <9 h)	Yes (IFG, pons, thalamus, frontal horn, and precentral gyrus after 24 h)	No	No	Peak glymphatic enhancement occurred within all CSF spaces and brain parenchyma (IFG) on T1WI after <9 h and 24 h	NA
	Zhou et al. (17)	Obj.	Yes (4^th^ (in 10 of 14), 3^rd^ (in 9 of 14), and lateral ventricles (in 9 of 14) after 4.5 h)	Yes (frontal horn (in 10 of 14), IFG (in 11 of 14), and precentral gyrus (in 12 of 14) after 15 h)	Yes (after 15 h in 9 of 14 individuals)	Yes (cervical LN after 39 h in 2 of 2^*^ individuals)	Peak glymphatic enhancement occurred within CSF space (fourth ventricle), brain parenchyma (precentral gyrus) on FLAIR after 4.5 h and 15 h	NA
Non-contrast MRI group	Kiviniemi et al. (11)	Obj.	Yes (periarterial)	Yes	Yes (perivenous)	No	NA	Glymphatic flows were demonstrated within the brain parenchyma on MREG (arterial pulsei induced glymphatic flow from the CSF spaces to the brain parenchyma and venous flow induce glymphatic flow from the brain parenchyma to MLV)
	Kuo et al. (12)	Subj.	No	No	Yes (alongside the SSS in 6 of 6 individuals)	No	NA	Glymphatic flows were demonstrated within MLV on TOF MRA (MLV had countercurrent flow to venous flow)
	Rajna et al. (15)	Obj.	Yes	Yes	Yes	No	NA	Glymphatic flows were demonstrated within the brain parenchyma on MREG (arterial pulse induced glymphatic flow from CSF spaces to the brain parenchyma)

*ACA, anterior cerebral artery; CSF, cerebrospinal fluid; fluid-attenuated inversion recovery (FLAIR); h, hours; IFG, inferior frontal gyrus; LN, lymph node; MCA, middle cerebral artery; min, minutes; MLV, meningeal lymphatic vessels; MREG, magnetic resonance encephalography; NA, not applicable; PCA, posterior cerebral artery; PHG, parahippocampal gyrus; pre., precontrast; Obj., objective signal quantification; SSS, superior sagittal sinus; sub., subjective visual assessment; T1WI, T1-weighted imaging; TOF MRA, time-of-flight MR angiography*.

* *The number of parent patients was not the same as the original enrolled number of patients because the peak enhancement can only be evaluated in the person who showed positive signal unit changes [18 (not 19) in the parenchyma and 17 (not 19) in the cervical LN in Eide et al. (9), and 2 (not 14) in the cervical LN in Zhou et al. (17)]*.

† *The increments of the globus pallidus-to-thalamus signal intensity ratio and the dentate nucleus-to-pons signal intensity ratio were evaluated between the initial unenhanced T1WI and control non-enhanced T1WI after performing contrast-enhanced MRC*.

In the non-contrast MRI group, two studies objectively measured flow-signal changes based on MRE (11, 15), whereas one study visualized this change on TOF-MRA (14). Arterial pulse induced centrifugal flow in the CSF space, whereas venous pulse induced centripetal flow in the MLV. Moreover, the flow of the MLV occurred countercurrent flow to the venous flow.

### Assessment of the Study Quality

**Figure 2:**
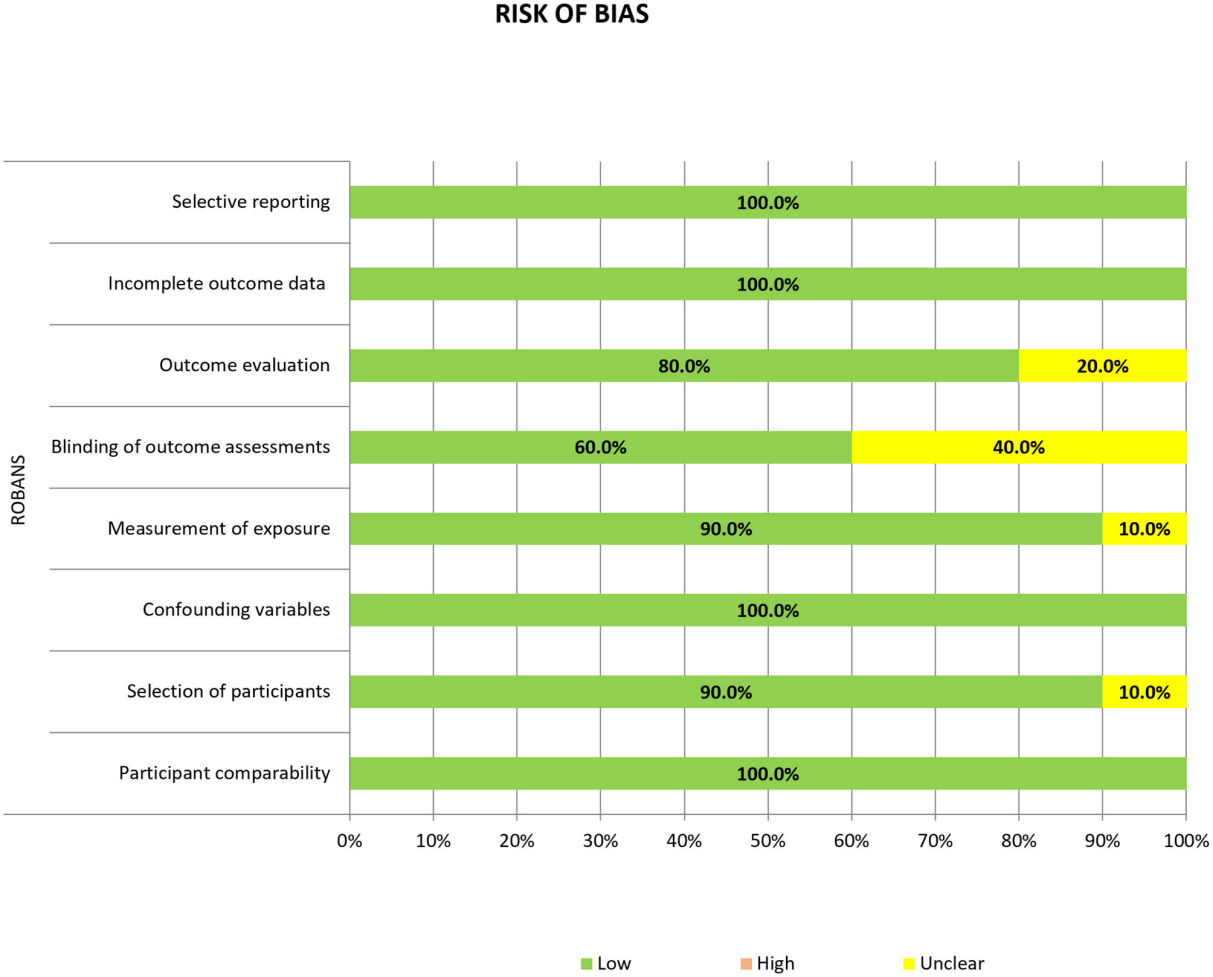
Risk of bias summary.

A quality assessment of the included studies was performed according to the criteria of the risk-of-bias assessment tool for non-standardized studies (RoBANS) and is summarized in Figure 2. All 10 studies showed a low risk of bias in term of participant comparability, confounding variables, incomplete outcome data, and selective reporting. However, one study showed an unclear risk of bias in the selection of participants, as the enrollment period of their retrospective study was not clearly defined (13). In the measurement of exposure, one study showed an unclear risk of bias because of the undescribed measurement methods (8). Four of the 10 studies showed an unclear risk of bias in the blinding of the outcome assessment due to unclear statements from the readers who underwent radiologic assessment (8, 11, 12, 15). Finally, in the outcome evaluation criterion, two studies showed an unclear risk of bias because they did not include a clear statement describing the imaging analysis (8, 12).

## Discussion

This systematic review evaluated and summarized the current research trends in MRI-based studies of the glymphatic hypothesis. The included studies mainly focused on the observation of contrast-enhancement or CSF flow changes within the anatomical structures that are considered to be the parts of the glymphatic system. Among the 10 included studies, seven and three were categorized into the contrast-enhanced MRI group and the non-contrast MRI group. To assess the CNS flow within glymphatic system, four studies from the contrast-enhanced MRI group performed a time-point dynamic observation of contrast enhancement within certain targeted locations and evaluated the peak enhancement time. In contrast, three studies from the non-contrast MRI group carried out TOF-MRA or phase-contrast MRE to visualize CSF flow. Within the system, arterial flow induced CSF influx from the subarachnoid space to the brain parenchyma, whereas venous flow induced ISF efflux from the brain parenchyma to the MLV.

Traditionally, the CNS has been characterized as lacking an anatomically defined lymphatic system to assist in CSF-ISF exchange (1). However, recent studies have suggested the presence of anatomical pathways that allow for flow exchange between the CSF and ISF spaces, and this CSF-ISF exchange should, theoretically, play a role in clearing ISF solute (4–6) from the CNS. This so-called “glymphatic” pathway have been observed in animal studies using fluorescent CSF tracers (4, 7, 51, 52), and researchers have shown that the tracers enter the brain parenchyma from the CSF space and are cleared through the MLV, after which they drain into the deep cervical LNs (4, 7). On the other hand, to assess the glymphatic flow in a human population, many studies have utilized various MRI techniques to observe CNS fluid enhancement and/or flow signal changes within the glymphatic system (8–17). In particular, recent studies have demonstrated an association between dysfunction of the glymphatic flow and various physiologic processes or neurologic diseases, including traumatic brain injury, Alzheimer's disease, and normal pressure hydrocephalus, and normal aging (2, 18–20). In this regard, acknowledging the presence of the glymphatic system is expected to provide a potential therapeutic target for treating many neurodegenerative diseases.

Nonetheless, glymphatic hypothesis is still on the debate. While the concept of glymphatic flow is mainly constituted by perivascular convective water influx, more recent studies have proposed different perspective on the CNS fluid movement that the solute transfer within the extracellular space may produce another momentum by diffusive conductivity without ISF flow. With such controversies, we believe that reviewing both supporting and conflicting hypotheses for CNS fluid flow is crucial to establish a generalized theory regarding CNS fluid dynamics. Thus, in our study, we aimed to assess the current state of the researches on the human glymphatic hypothesis first. Through our systematic review, we sought to improve the robustness of the methodology, and to lay the groundwork for future development of a standardized imaging protocol for *in vivo* CNS fluid study. The main strength of this systematic review is that this is the first to encompass imaging studies that demonstrated the presence of CNS flow following the glymphatic hypothesis in a human population without possessing any prior and present CNS pathology.

To dilate on the glymphatic hypothesis, the function of the glymphatic system has been described as a mean of facilitating ISF exchange in the CNS system, and many previous studies (5–7) have reported that the glymphatic flow begins in the CSF space and sequentially flows into the brain parenchyma, and this fluid exchange allows for efflux into the MLV and cervical LNs (Supplementary Figures 1A,B). Accordingly, our included studies in the contrast-enhanced MRI group revealed the peak enhancement time occurring first in the CSF space, then in the brain parenchyma and MLV, and finally in the cervical LNs. This peak enhancement pattern was similar to that has been described in previous studies of the glymphatic system and consistent with the flow patterns revealed by radiotracer examinations in animal (6, 7), presenting possibility of visualizing glymphatic flow on the MRI. Moreover, the results of the studies in the non-contrast MRI group revealed similar CSF flow patterns proposed in the glymphatic system. They demonstrated that arterial pulse induces centrifugal flow in the CSF space, whereas venous pulse induces centripetal flow in the MLV. In addition, the flow of the MLV runs countercurrent to the venous flow. Similar to the change in the peak enhancement on contrast-enhanced MRI, the non-contrast MRI studies revealed that the flow change began in the CSF space and was extended toward the brain parenchyma and MVL. Therefore, these results may support the glymphatic hypothesis in a human population. This demonstration of the glymphatic flow on both contrast-enhanced and non-contrast MRI can have significant clinical value, as such imaging can detect of the glymphatic dysfunction, and can disclose the underlying pathophysiology of certain neurodegenerative diseases, including Alzheimer's disease and normal pressure hydrocephalus (53–55). Therefore, we believe that this systematic review could provide a basis for the future studies linking glymphatic dysfunction and neurodegenerative diseases.

According to the results from the studies in our review, we can surmise that that contrast-enhanced MRI can be superior to non-contrast flow-sensitive MRI for glymphatic imaging, as the analysis of changes in CSF flow on non-contrast MRI is subjective, whereas contrast-enhanced MRI can provide objective measurements of the degree of glymphatic flow changes as well as subjective analysis. Therefore, we recommend contrast-enhanced T1-weighted MRI using a gadolinium-based contrast agent for dedicated the glymphatic imaging. Among the studies comprising the contrast-enhanced MRI group, most, except for that of Absinta et al. and Naganawa et al. (8, 13), used intrathecal rather than intravenous injection of contrast agents. Thus, as to the glymphatic imaging using intrathecal contrast injection, we can recommend the following time schedule for measuring the peak enhancement within specific components of the glymphatic system with a high degree of confidence: for the CSF, within 4–6 h; for the parenchyma and MLV, within 15–48 h, and for the cervical LNs, after more than 24 h. On the other hand, it is difficult to make recommendation for the standardized imaging protocols such as appropriate time frame or proper region-of-interest allocation with the use of intravenous injection of contrast agents since there was only one relevant study based on this route of administration was performed (13). However, we must consider the fact that intrathecal injection of contrast agents can be associated with several drawbacks, including neurotoxicity, which can induce chemical meningitis, high degree of procedural invasiveness, infection, hemorrhage, nerve injury, radiation exposure, and/or hypersensitivity reaction (56–59). Due to the above safety issue and high risk of the complication, intrathecal injection of the gadolinium-based contrast agents is not allowed in many countries, particularly for healthy human subjects. Therefore, intrathecal contrast injection-based glymphatic MRI can be limited in its usage, and accordingly, the randomized controlled study can hardly be performed due to ethical constraints (60). Considering this, we should suggest that the route of contrast agent administration for the glymphatic imaging must be carefully selected according to the patient's condition, medical history, and age. Since the intravenous injection of contrast agent is more accessible than the intrathecal route in clinical practice, future studies using intravenous contrast injection are expected to broaden the application of the glymphatic imaging in the clinical setting and assist in developing the standardized imaging protocols. In addition, we expect that advanced MRI techniques to reduce scan time such as compressed sensing can be adopted in the glymphatic imaging using intravenous contrast injection, and allow clinically feasible MRI protocol for human population while maintaining or even improving imaging quality.

Recently, a method of non-invasive glymphatic assessment has been introduced to overcome the drawbacks of contrast-enhanced MRI. For instance, Taoka et al. have utilized diffusion tensor imaging (DTI) and developed a method called “Diffusion Tensor Image Analysis aLong the Perivascular Space (DTI-ALPS)” to evaluate the perivascular diffusivity reflecting glymphatic function (61–64). This DTI-based method has effectively shown the altered glymphatic perivascular flow in many neurodegenerative diseases, and thus, have shown the potential to be a promising clinical application due to its non-invasiveness (64). Nonetheless, DTI-based glymphatic studies could not be included in our current systematic review, since all published studies were based on the diseased population, while our review was focused on the MRI-based demonstration of the glymphatic flow in a normal population. We are planning future reviews dealing with the CNS fluid dynamics within the population with neurodegenerative disease, and the corresponding inclusion of the DTI-based glymphatic imaging studies is expected to contribute to set the recommendations for the imaging protocol.

Our study had several limitations. First, since we aimed to investigate imaging studies on the glymphatic hypothesis in human participants without any underlying CNS pathology, we could only include a small number of studies. Most of the published glymphatic imaging studies are based on the intrathecal contrast injection, but as aforementioned, this procedure is note routinely performed in healthy subjects due to high risk (56–59). Second, the number of the included studies using intravenous contrast injection was too small. We included two studies in which the contrast agent was injected intravenously to assess the glymphatic system, and all of them evaluated the contrast enhancement parameter at a single time point and a location. Therefore, it is difficult to draw conclusions about the timing and the pattern of the enhancement reflecting glymphatic flow in these studies. Since intravenous contrast injection is safer than intrathecal injection (57, 59), future larger studies using the former will contribute to the ever-growing body of the glymphatic research. Third, although we attempted to include studies based on the normal healthy population without significant CNS disorders. We included such patients, because all of these patients did not have any underlying CNS diseases, but their CSF leakage was induced by minor trauma and their endolymphatic hydrops was peripheral vestibular disease separated from CNS disease. Therefore, we believe that our inclusion criteria made successful inclusion of the human without CNS disorder. Fourth, as previously described, we did not include studies that used DTI techniques in the current review. Although promising and feasible DTI-based glymphatic imaging has been performed only on the patients with CNS disorders (63), which is beyond the scope of our present review focusing on the normal glymphatic flow imaging in the human population without significant CNS disorders. Future reviews including studies that have employed DTI techniques and those that have targeted patients with CNS disorders will add much to our current study results. Fifth, there was no study that distinguished perivenous and non-perivenous spaces. The glymphatic hypothesis suggests that periarterial influx is followed by perivenous efflux (65). Although most studies in the contrast-enhanced MRI group evaluated presence of enhancement within the brain parenchyma, the increment of signal intensity within the brain parenchyma may reflect CSF inflow rather than direct periarterial influx. Further studies developing imaging protocols that distinguish perivenous and non-perivenous spaces will be needed.

The present systematic review evaluated the results of the published studies on the glymphatic MRI in terms of the imaging sequence, the imaging protocol including contrast agent injection protocol and acquisition time points on dynamic contrast-enhanced scanning, and the targeted anatomical structures for measuring the glymphatic flow. Our findings could enhance the understanding of the glymphatic hypothesis for the CNS fluid movement in human and help generate the standardized MRI protocols for measuring glymphatic flow. Based on this review, we may recommend contrast-enhanced T1-weighted MRI over non-contrast flow-sensitive MRI for the glymphatic imaging, but with cautious use of the intrathecal contrast injection. We believe that future studies that can link neurodegenerative diseases and glymphatic function and that can review the counter diffusivity theory for the CNS fluid dynamic can be built on the basis of our current study.

## Data Availability Statement

The raw data supporting the conclusions of this article will be made available by the authors, without undue reservation.

## Author Contributions

ML and SC contributed to conception, design of the study, and organized the database. SC performed the statistical analysis. ML wrote the first draft of the manuscript. ML, SC, and YB wrote sections of the manuscript. All authors contributed to manuscript revision, read, and approved the submitted version.

## Funding

This work was supported by the National Research Foundation of Korea (NRF) grant funded by the Korea government (MSIT) (No. 2019R1F1A1063771) and grant No. 02-2021-0015 from the SNUBH Research Fund.

## Conflict of Interest

The authors declare that the research was conducted in the absence of any commercial or financial relationships that could be construed as a potential conflict of interest.

## Publisher's Note

All claims expressed in this article are solely those of the authors and do not necessarily represent those of their affiliated organizations, or those of the publisher, the editors and the reviewers. Any product that may be evaluated in this article, or claim that may be made by its manufacturer, is not guaranteed or endorsed by the publisher.

## Supplementary Material

Supplementary Figure S1 Schematic illustration of the glymphatic system. **(A)** The glymphatic system starts within the CSF space along the periarterial area and then is transported into the brain parenchyma. The exchanged fluid between the CSF and interstitial fluid (ISF) flows to the meningeal lymphatic vessel (MLV). **(B)** Close-up view of CSF and ISF exchange within the brain parenchyma. SSS, superior sagittal sinus.
