# Supplementary figures and images for: MRI-Based Demonstration of the Normal Glymphatic System in a Human Population: A Systematic Review
Source: Front Neurol. 2022 May 25;13:827398. doi: 10.3389/fneur.2022.827398 (PMC9174517; doi:10.3389/fneur.2022.827398)

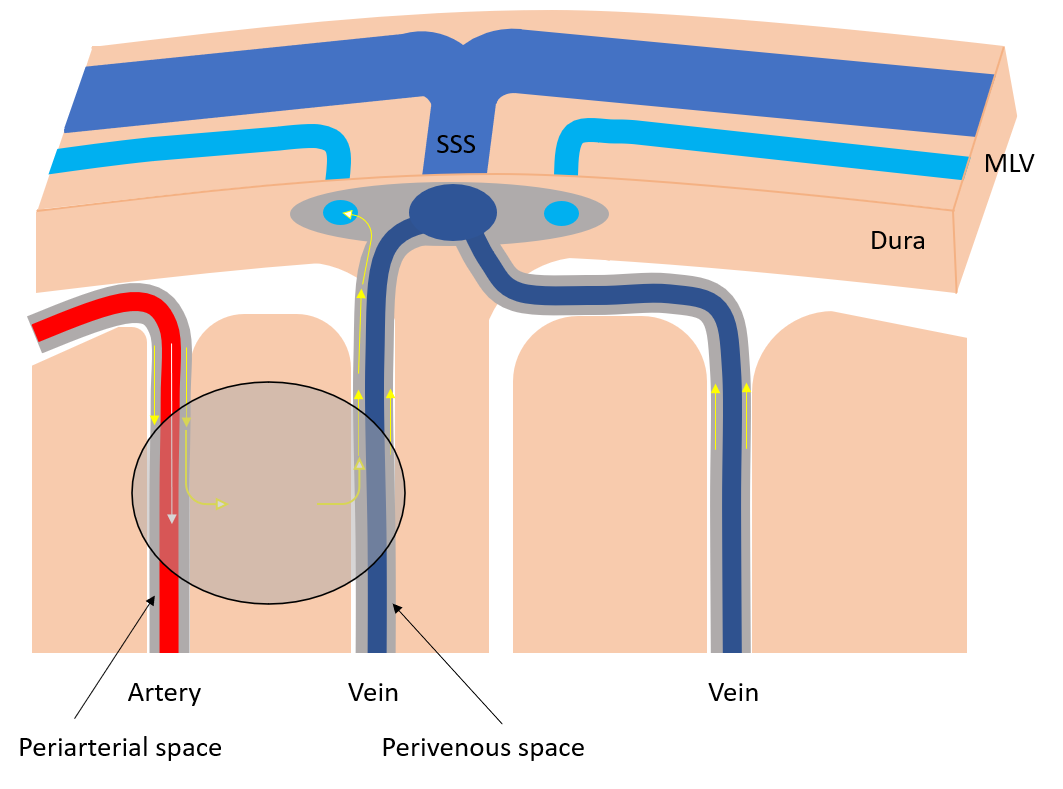

Supplement: Supplementary file 1 [file Image_1.TIF]

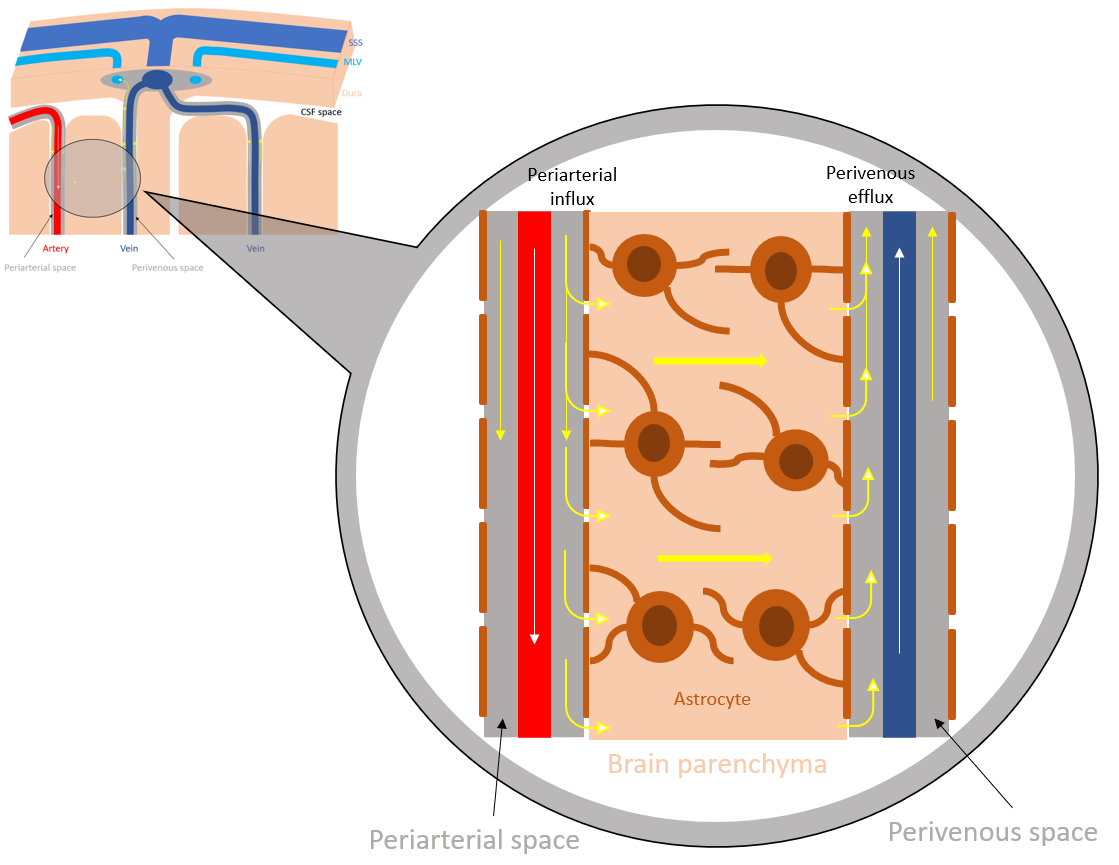

Supplement: Supplementary Figure S1 — Schematic illustration of the glymphatic system. (A) The glymphatic system starts within the CSF space along the periarterial area and then is transported into the brain parenchyma. The exchanged fluid between the CSF and interstitial fluid (ISF) flows to the meningeal lymphatic vessel (MLV). (B) Close-up view of CSF and ISF exchange within the brain parenchyma. SSS, superior sagittal sinus. [file Image_2.TIF]
